# Trends in immune cell profiles of osteomyelitis: a clinical study supported by Mendelian randomization analysis

**DOI:** 10.3389/fmed.2025.1669180

**Published:** 2025-09-29

**Authors:** Zemin Liu, Guochao Jin, Yanming Zhi, Shengxian Ge, Dong Wang, Juan Li, Yan Li, Yonghong Zhang

**Affiliations:** ^1^Department of Orthopaedics, Second Hospital of Shanxi Medical University, Taiyuan, Shanxi, China; ^2^Second Clinical Medical College, Shanxi Medical University, Taiyuan, Shanxi, China; ^3^Department of Ultrasound, Second Hospital of Shanxi Medical University, Taiyuan, China

**Keywords:** osteomyelitis, immune cells, lymphocytes, neutrophils, Mendelian randomization, peripheral blood analysis

## Abstract

**Background:**

Osteomyelitis, a persistent inflammatory bone disease, is only partially responsive to conventional antibiotics and surgery. Some patients experience poor outcomes and relapse. Recently, immunotherapy has emerged as a promising treatment strategy. This study combined clinical research with Mendelian randomization analysis to explore the interaction and relationship between immune cells and osteomyelitis, aiming to offer novel therapeutic insights.

**Methods:**

We retrospectively analyzed blood test data from patients admitted between July 1, 2023, and December 31, 2024, including those undergoing internal fixator removal and those with osteomyelitis. Based on the bacterial culture results, patients with osteomyelitis were categorized into four subgroups: Gram-positive, Gram-negative, mixed infections, and culture-negative. The impact of bacterial infections on immune cells was assessed, and two-sample Mendelian randomization was applied to evaluate the bidirectional causality between immune cells and osteomyelitis. Causal effects were primarily estimated using inverse-variance weighted and weighted-median methods, with F-statistic calculations and sensitivity analyses conducted to bolster the credibility of the results.

**Results:**

Compared to the internal fixator removal group, patients with osteomyelitis exhibited significantly reduced neutrophils but elevated lymphocytes, eosinophils, and basophils. Subgroup analysis revealed significantly increased lymphocyte counts and decreased neutrophil counts across all subgroups, except for the mixed infection group. Two-sample Mendelian randomization indicated a causal link between circulating lymphocytes and osteomyelitis risk [odds ratio (OR): 1.203; 95% confidence interval (CI): 1.064–1.362; *p* = 0.003], supported by weighted median analysis (OR: 1.273; 95% CI: 1.047–1.549; *p* = 0.016). Sensitivity analyses confirmed the robustness and reliability of the results with no significant pleiotropy or heterogeneity.

**Conclusion:**

Osteomyelitis was associated with significant alterations in peripheral immune cell counts, particularly lymphocyte counts, which showed a strong positive correlation with the disease risk. These findings pave the way for future immunotherapeutic approaches for the treatment of osteomyelitis.

## 1 Introduction

Osteomyelitis, an inflammatory bone disease caused by pathogenic infections, involves complex interactions between pathogens, host immune responses, and the local microenvironment (1–3). Despite recent advances in diagnostic and therapeutic techniques, osteomyelitis remains a challenging disease to treat because of biofilm formation and pathogen immune evasion. However, it is typically characterized by a long treatment duration, high recurrence rate, and significant disability, which impose a heavy burden on patients and society (4, 5). In recent years, with a deeper understanding of the pathogenesis of osteomyelitis, new therapeutic approaches such as vaccines and immunotherapies have been proposed as adjuncts to traditional surgical and antibiotic treatments (6–8). Previous studies on laboratory indicators of osteomyelitis have mainly focused on evaluating C-reactive protein, erythrocyte sedimentation rate, procalcitonin, and total white blood cell count. However, the specific changes in individual immune cell subsets during osteomyelitis pathogenesis remain unclear (9–11).

The immune system plays a central role in resisting pathogenic invasion and maintaining tissue homeostasis. As key components of the immune system, immune cells play a dual role in the occurrence and development of osteomyelitis. Conversely, innate immune cells like neutrophils and macrophages can quickly identify and eliminate pathogens, playing a crucial defensive role in the early stages of infection (12). In contrast, over-activated immune cells release a large number of inflammatory factors, leading to tissue damage and bone destruction (13). Bone tissue homeostasis mainly depends on the balance between osteoblasts and osteoclasts. A major pathological feature of osteomyelitis is an imbalance in bone homeostasis, metabolic disorders, and subsequent bone damage, all of which seriously affect bone healing. The increased differentiation of immune cells (such as monocytes) into osteoclasts promotes bone loss in patients with osteomyelitis (14). The exact interactions between immune cells and osteomyelitis remain unclear, highlighting the need for further research.

Clinical studies using methods such as propensity score matching (PSM) to reduce confounding factors still have biases, confusion, and sample size issues that limit their ability to establish causal relationships. Mendelian randomization (MR) uses germline genetic variants as instrumental variables to move beyond observational associations and generate testable hypotheses about potential causal relationships.

Our study combined clinical research with MR analysis to explore immune cell changes and their causal relationships in osteomyelitis. This clinical section examines the characteristics of different immune cells in patients with osteomyelitis. The MR analysis investigates the bidirectional causality between immune cells and osteomyelitis, offering a theoretical basis and direction for future immunotherapy research.

## 2 Methods

### 2.1 Study design

**Figure 1:**
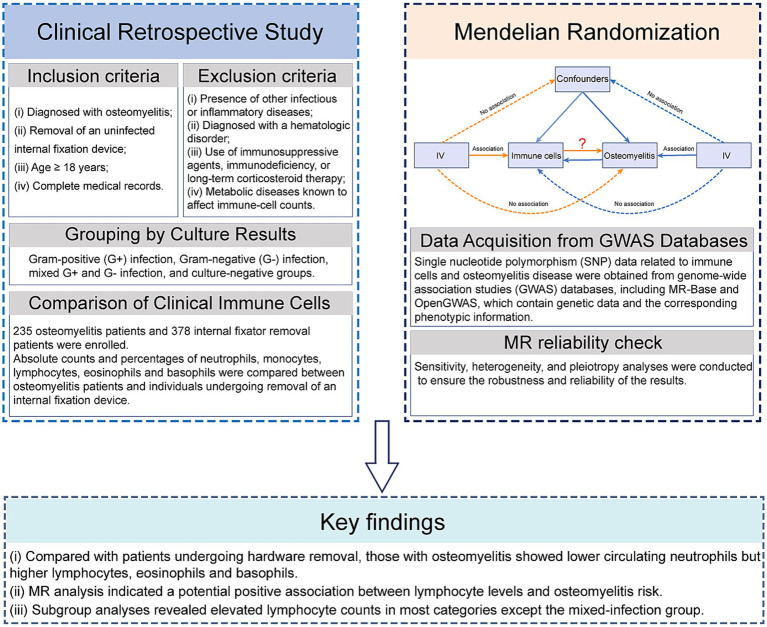
Clinical and MR study design flowchart.

A retrospective analysis was conducted on data from patients with osteomyelitis and those undergoing internal fixator removal who were admitted to the Second Hospital of Shanxi Medical University between January 1, 2021, and December 31, 2024. The collected variables included sex, age, height, weight, smoking history, diabetic status, time of onset, symptoms, signs, imaging findings, and blood test results. This study was approved by the Ethics Committee of the Second Hospital of Shanxi Medical University (approval no. 2025-YX-226). Patients who underwent internal fixator removal served as the control group, whereas patients with osteomyelitis constituted the experimental group. Patients with osteomyelitis were categorized into four subgroups based on the bacterial culture results: Gram-positive (G+) infection, Gram-negative (G−) infection, mixed G+ and G− infection, and culture-negative groups (Figure 1).

### 2.2 Inclusion and exclusion criteria

The inclusion criteria were as follows: (i) Patients clinically diagnosed with osteomyelitis via imaging, laboratory tests (e.g., C-reactive protein and blood culture), and clinical presentation. (ii) Patients with closed fractures undergoing internal fixator removal without infection. (iii) Patients aged ≥ 18 years. (iv) Patients with complete medical records.

The exclusion Criteria were as follows: (i) Patients with other infections or inflammatory diseases. (ii) Patients with hematological diseases. (iii) Patients on immunosuppressants, with immunodeficiency (e.g., AIDS) or on long-term corticosteroids. (iv) Patients with metabolic diseases that affect immune cell counts (e.g., hyperthyroidism). (v) Patients with incomplete medical records (Figure 1).

### 2.3 MR analysis methods

Single nucleotide polymorphism (SNP) data related to immune cells and osteomyelitis disease were obtained from genome-wide association studies (GWAS) databases, including MR-Base and OpenGWAS, which contain genetic data and the corresponding phenotypic information. SNPs associated with immune cell counts or functions were selected as instrumental variables (IVs) based on the following criteria: (i) Strong association between IVs and exposure: Exposure-related SNPs were identified by a screening with a specific *p*-value threshold. (ii) No direct association between IVs and outcomes: Outcome information was extracted for the IVs obtained and SNPs related to the outcome were excluded (*p* < 0.05). (iii) No association between IVs and confounding factors: Traits related to the IVs were retrieved from databases such as PubMed, OpenGWAS, GWAS Catalog, and VannoPORtal, and IVs potentially associated with confounding factors were excluded. SNPs in linkage disequilibrium were removed from the filtered IVs, and the effect alleles and effect sizes of the IVs were unified to ensure consistency for subsequent MR analysis.

MR analysis was performed using the TwoSampleMR package in the R software. The analysis steps were as follows: (i) Harmonization of the extracted exposure and outcome data to ensure consistency in the effect alleles and effect sizes of the instrumental variables. (ii) We performed MR with five complementary estimators—inverse variance weighting (IVW), weighted median (WM), MR-Egger, weighted mode and simple mode. The IVW and WM were used for primary analysis, while additional methods were employed to address horizontal pleiotropy and ensure robust findings. (iii) The F-statistic was calculated for each IVs to avoid bias from weak instruments. (iv) Sensitivity, heterogeneity, and pleiotropy analyses were conducted to ensure the robustness and reliability of the results (Figure 1).

### 2.4 Statistical analysis

Data were organized and analyzed using SPSS 22.0. When the baseline data were imbalanced between the groups, PSM was applied. Using a 1:1 nearest-neighbor matching method, patients with osteomyelitis were matched with patients who underwent internal fixator removal based on variables such as sex, age, height, weight, smoking history, and diabetes status, with a matching tolerance of 0.02. For unmatched data, normally distributed continuous variables were compared using independent-sample t-tests, non-normally distributed variables were analyzed using Mann–Whitney U tests, and categorical variables were analyzed using chi-square tests. For PSM-matched data, paired t-tests were used for normally distributed continuous variables, Wilcoxon tests for non-normally distributed continuous variables, and McNemar’s tests for categorical variables. Normally distributed continuous data are presented as mean ± standard deviation, non-normally distributed data as median and interquartile range, and categorical data as counts. Statistical significance was set at *p* < 0.05.

## 3 Results

### 3.1 Patient baseline characteristics and data processing

Overall, 235 osteomyelitis patients and 378 internal fixator removal patients were enrolled. Five patients had fungal infections, a subgroup not separately categorized owing to small size. Baseline data were balanced using PSM, yielding 235 matched pairs for overall osteomyelitis analysis (Supplementary Table 1). The G+ and internal fixator removal groups had comparable baseline data (Supplementary Table 2). The G− group underwent PSM with a tolerance of 0.02, successfully matching 29 pairs (Supplementary Table 3). Owing to significant sample size differences, the mixed infection group was matched with the internal fixator removal group using PSM, resulting in 19 pairs (Supplementary Table 4). The culture-negative group was matched with the internal fixator removal group using PSM with a tolerance of 0.02, yielding 69 matched pairs (Supplementary Table 5).

### 3.2 Clinical findings: immune cell changes in patients with osteomyelitis

Significant differences in immune cell profiles were observed between the osteomyelitis and internal fixator removal groups. Compared with the internal fixator removal group, patients with osteomyelitis exhibited significantly lower counts of white blood cells, neutrophils, monocytes and reduced percentages of neutrophils. In contrast, the counts and percentages of lymphocytes, eosinophils, and basophils were significantly higher in patients with osteomyelitis. Additionally, the percentage of monocytes was significantly higher in the osteomyelitis group (Table 1).

**Table 1:** Comparison of circulating immune cell profiles between patients with overall osteomyelitis and implant-removal.

Items	WBC#	Neu#	Mono#	Lym#	Eo#	Baso#	Neu%	Mono%	Lym%	Eo%	Baso%
IR	8.38 ± 2.38	6.02 ± 2.13	0.54 [0.42, 0.71]	1.67 ± 0.63	0.07 [0.03, 0.12]	0.02 [0.01, 0.03]	71.4 [62.95, 77.35]	7.04 ± 1.93	20.91 ± 7.95	0.9 [0.3, 1.8]	0.2 [0.1, 0.4]
OM	6.88 ± 2.19	4.25 ± 1.93	0.50 [0.39, 0.60]	1.92 ± 0.63	0.13 [0.07, 0.20]	0.03 [0.02, 0.04]	60.2 [54.2, 66.3]	7.54 ± 1.91	28.57 ± 8.68	1.9 [1.2,3.0]	0.5 [0.3, 0.6]
*p*	<0.001	<0.001	<0.001	<0.001	<0.001	<0.001	<0.001	0.006	<0.001	<0.001	<0.001

IR, implant-removal; OM, osteomyelitis; WBC, White blood cell; Neu, Neutrophil; Mono, Monocyte; Lym, Lymphocyte; Eo, Eosinophil; Baso, Basophil; #, count (10^9^/L); %, Percentage.

Compared to the internal fixator group, the G+ osteomyelitis group showed reduced white blood cell counts, neutrophil counts, and neutrophil percentages, while lymphocyte, eosinophil, and basophil counts and percentages were increased. Monocyte counts showed no significant differences, but the monocyte percentage was higher in the osteomyelitis group (Table 2).

**Table 2:** Comparison of circulating immune cell profiles between patients with Gram-positive monomicrobial osteomyelitis and implant-removal.

Items	WBC#	Neu#	Mono#	Lym#	Eo#	Baso#	Neu%	Mono%	Lym%	Eo%	Baso%
IR	7.99 [6.42, 9.72]	5.54 [4.19, 7.2]	0.52 [0.4, 0.68]	1.58 [1.21, 2.07]	0.06 [0.03, 0.11]	0.02 [0.01, 0.03]	70.02 ± 9.7	6.9 [5.575, 8.2]	20.75 [14.95, 27.92]	0.8 [0.3, 1.6]	0.2 [0.1, 0.4]
OM	6.77 [5.54, 8.2]	4.19 [3.13, 5.11]	0.51 [0.41, 0.66]	1.88 [1.54, 2.36]	0.12 [0.07, 0.2]	0.03 [0.02, 0.05]	60.67 ± 9.16	7.8 [6.45, 8.65]	30 [22.6, 33.85]	1.8 [1.1, 2.8]	0.5 [0.3, 0.7]
*p*	<0.001	<0.001	0.596	<0.001	<0.001	<0.001	<0.001	<0.001	<0.001	<0.001	<0.001

IR, implant-removal; OM, osteomyelitis; WBC, White blood cell; Neu, Neutrophil; Mono, Monocyte; Lym, Lymphocyte; Eo, Eosinophil; Baso, Basophil; #, count (10^9^/L); %, Percentage.

In the G− infection osteomyelitis group, compared with the internal fixator removal group, there was a significant decrease in the white blood cell count, neutrophil count, and neutrophil percentage, and a significant increase in the basophil count, basophil percentage, and lymphocyte percentage. However, no significant differences were found in monocyte count, monocyte percentage, lymphocyte count, eosinophil count, or eosinophil percentage between the two groups (Table 3).

**Table 3:** Comparison of circulating immune cell profiles between patients with Gram-negative monomicrobial osteomyelitis and implant-removal.

Items	WBC#	Neu#	Mono#	Lym#	Eo#	Baso#	Neu%	Mono%	Lym%	Eo%	Baso%
IR	7.95 ± 1.75	5.66 ± 1.53	0.56 ± 0.2	1.58 ± 0.57	0.13 ± 0.13	0.02 ± 0.01	70.78 ± 8.19	7.09 ± 2.16	21.15 ± 8.11	1.77 ± 1.75	0.25 ± 0.14
OM	7.1 ± 2.19	4.60 ± 2.08	0.49 ± 0.14	1.84 ± 0.6	0.15 ± 0.11	0.03 ± 0.01	62.39 ± 10.78	7.25 ± 2.02	27.71 ± 9.45	2.20 ± 1.52	0.42 ± 0.22
*p*	0.070	0.030	0.127	0.104	0.623	0.037	0.006	0.745	0.019	0.383	0.004

IR, implant-removal; OM, osteomyelitis; WBC, White blood cell; Neu, Neutrophil; Mono, Monocyte; Lym, Lymphocyte; Eo, Eosinophil; Baso, Basophil; #, count (10^9^/L); %, Percentage.

Compared with the internal fixator group, neutrophil counts were significantly reduced in both the G+ and G− mixed infection osteomyelitis groups, whereas basophil percentages were elevated. However, no significant differences were found in white blood cell counts, monocyte counts and percentages, lymphocyte counts, neutrophil percentages, eosinophil counts and percentages, basophil counts, or lymphocyte percentages between the two groups (Table 4).

**Table 4:** Comparison of circulating immune-cell profiles between patients with polymicrobial (Gram-positive and Gram-negative) osteomyelitis and implant-removal.

Items	WBC#	Neu#	Mono#	Lym#	Eo#	Baso#	Neu%	Mono%	Lym%	Eo%	Baso%
IR	7.7 ± 1.45	5.34 [4.21, 6.36]	0.52 ± 0.18	1.6 ± 0.59	0.13 ± 0.11	0.02 ± 0.01	70.31 ± 8.92	6.1 [4.6, 8.3]	20.79 ± 6.97	1.75 ± 1.63	0.3 [0.2, 0.3]
OM	6.73 ± 2.32	3.63 [2.99, 5.08]	0.45 ± 0.14	1.65 ± 0.62	0.15 ± 0.1	0.03 ± 0.02	64.29 ± 10.17	6.9 [6.3, 7.6]	26.21 ± 9.88	2.22 ± 1.48	0.4 [0.3, 0.6]
*p*	0.121	0.009	0.257	0.823	0.645	0.056	0.055	0.643	0.063	0.435	0.003

IR, implant-removal; OM, osteomyelitis; WBC, White blood cell; Neu, Neutrophil; Mono, Monocyte; Lym, Lymphocyte; Eo, Eosinophil; Baso, Basophil; #, count (10^9^/L); %, Percentage.

White blood cell counts, neutrophil counts and percentages, and monocyte counts were significantly lower in the bacterial culture-negative osteomyelitis group than in the internal fixator removal group (Table 5). No significant difference was observed in the monocyte percentage between the two groups.

**Table 5:** Comparison of circulating immune-cell profiles between culture-negative osteomyelitis patients and implant-removal.

Items	WBC#	Neu#	Mono#	Lym#	Eo#	Baso#	Neu%	Mono%	Lym%	Eo%	Baso%
IR	8.34 ± 2.28	6.01 ± 2.11	0.57 ± 0.21	1.64 ± 0.61	0.08 [0.04, 0.12]	0.02 [0.01, 0.03]	71.9 [65.7, 76]	6.90 ± 2.05	20 [14.45, 27.4]	0.9 [0.5, 1.6]	0.24 ± 0.13
OM	6.57 ± 1.99	3.94 ± 1.55	0.48 ± 0.15	2 ± 0.68	0.13 [0.08, 0.21]	0.03 [0.02, 0.05]	60.4 [53.65, 63.65]	7.39 ± 1.96	30.3 [23.8, 33.85]	2.1 [1.15, 3.2]	0.5 ± 0.26
*p*	<0.001	<0.001	0.006	0.001	0.002	<0.001	<0.001	0.142	<0.001	<0.001	<0.001

IR, implant-removal; OM, osteomyelitis; WBC, White blood cell; Neu, Neutrophil; Mono, Monocyte; Lym, Lymphocyte; Eo, Eosinophil; Baso, Basophil; #, count (10^9^/L); %, Percentage.

### 3.3 MR analysis reveals a causal relationship between lymphocytes and osteomyelitis

Following the selection criteria outlined in the Methods section, all included SNPs demonstrated strong IVs effects (F-statistic > 10). The details of the IVs used in the MR analysis are provided in Supplementary Table 6.

#### 3.3.1 Effect of circulating lymphocytes on osteomyelitis

**Figure 2:**
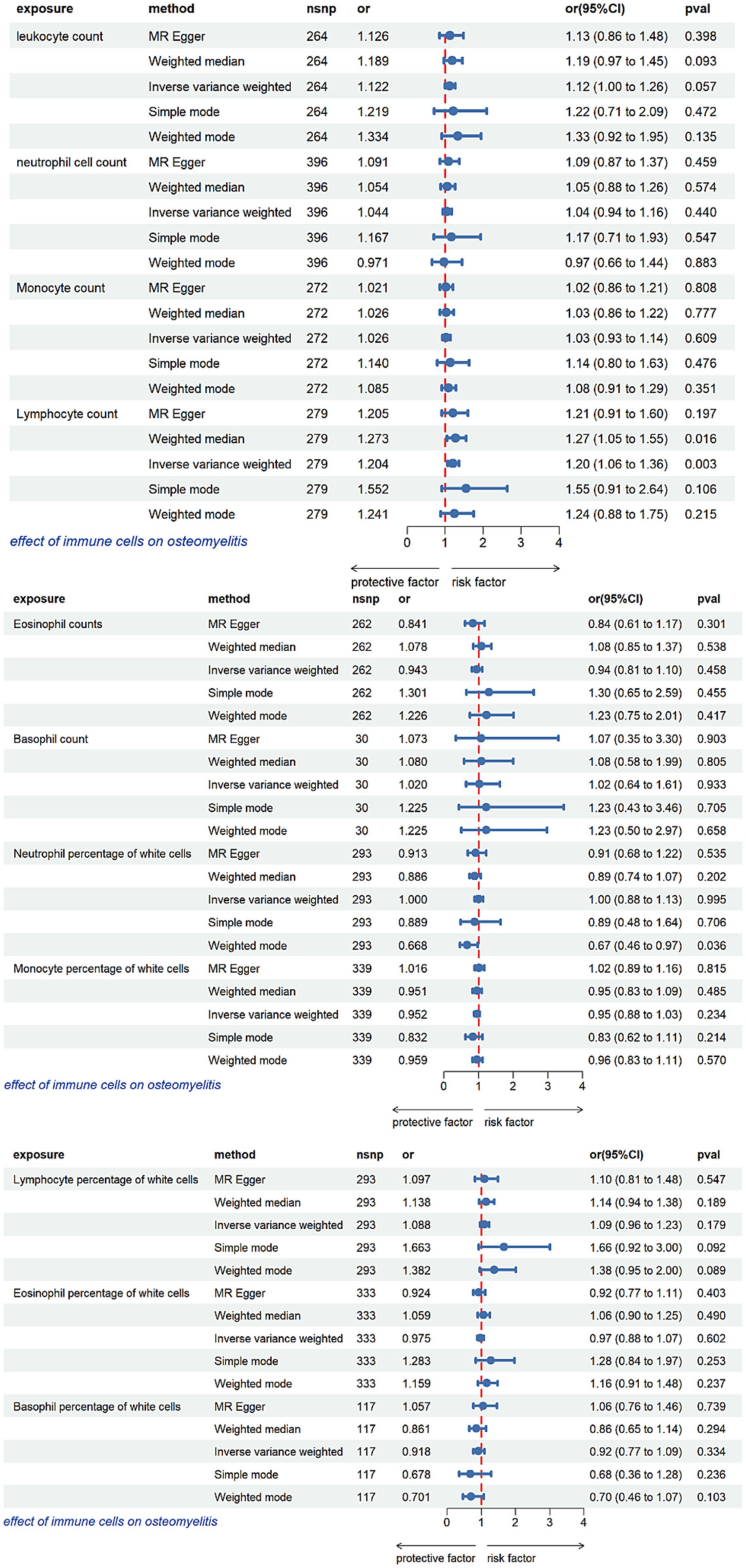
Mendelian randomization analysis of the causal impact of circulating immune cells on osteomyelitis.

The IVW method revealed a significant association between an increased circulating lymphocyte count and a higher risk of osteomyelitis [odds ratio (OR): 1.203; 95% confidence interval (CI): 1.064–1.362; *p* = 0.003], indicating a positive correlation between these variables (Figure 2). This trend was consistent with the WM analysis (OR, 1.273; 95% CI, 1.047–1.549; *p* = 0.016). Although statistically non-significant (*p* > 0.05), the results from the other three MR methods (MR-Egger regression, weighted mode, and simple mode) yielded OR estimates > 1, aligning with the same directional effect (Figure 2).

**Figure 3:**
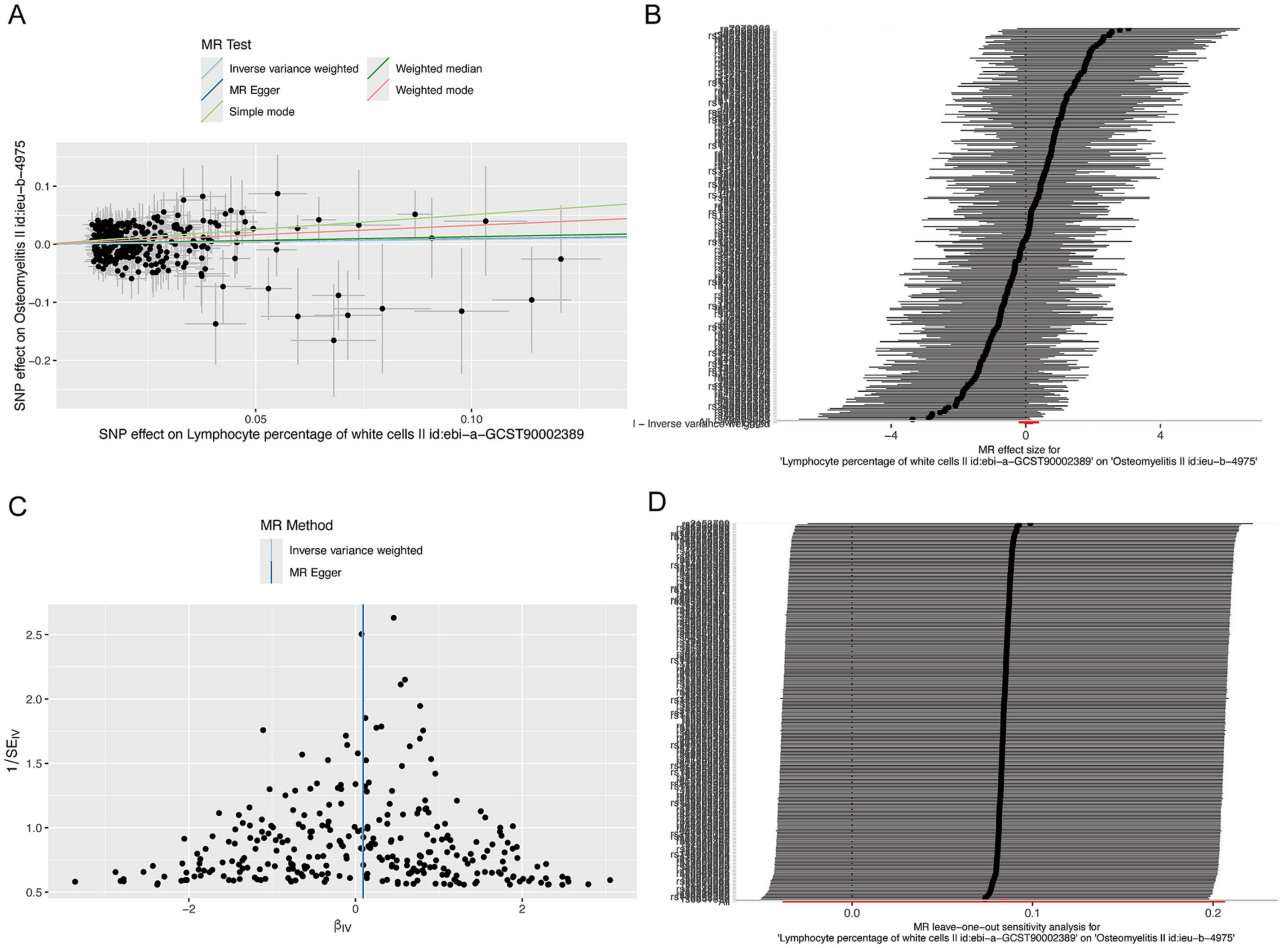
Positive causal association between circulating lymphocytes and osteomyelitis. **(A)** Scatter plot illustrating the relationships of individual SNPs with circulating lymphocytes and osteomyelitis. **(B)** Forest plot presenting the pooled causal estimate with 95% confidence intervals for the overall causal effect. **(C)** Funnel plot assessing the heterogeneity among instrumental SNPs. **(D)** Leave-one-out analysis evaluating the influence of each SNP on the Mendelian randomization estimates.

Scatter plots visually confirmed the positive association between circulating lymphocytes and osteomyelitis (Figure 3A). Forest plots of the combined effect estimates and CIs further supported this positive relationship (Figure 3B). No significant pleiotropy was detected by the MR-Egger intercept method. Heterogeneity analyses (the IVW and MR-Egger methods) revealed no substantial heterogeneity. Funnel plot symmetry indicated the absence of directional pleiotropy or significant bias in effect estimates (Figure 3C). Leave-one-out sensitivity analysis demonstrated the stability and robustness of the causal effect, as no single IV significantly altered the overall estimate upon removal (Figure 3D).

MR analyses using IVW, WM, MR-Egger regression, weighted mode, and simple mode methods revealed no evidence of a potential causal relationship between the risk of osteomyelitis and absolute counts or relative percentages of leukocytes, neutrophils, monocytes, eosinophils, or basophils. No significant horizontal pleiotropy was detected using the MR-Egger intercept test (*p* > 0.05). Furthermore, heterogeneity analyses using both IVW and MR-Egger methods showed no substantial heterogeneity (*p* > 0.05).

#### 3.3.2 The effect of osteomyelitis on circulating immune cells

Following the selection criteria detailed in the Methods section, all IVs used in the analysis exhibited F-statistics > 10, indicating a low likelihood of weak instrumental bias. A complete list of the IVs used in the MR analysis is provided in Supplementary Table 7.

**Figure 4:**
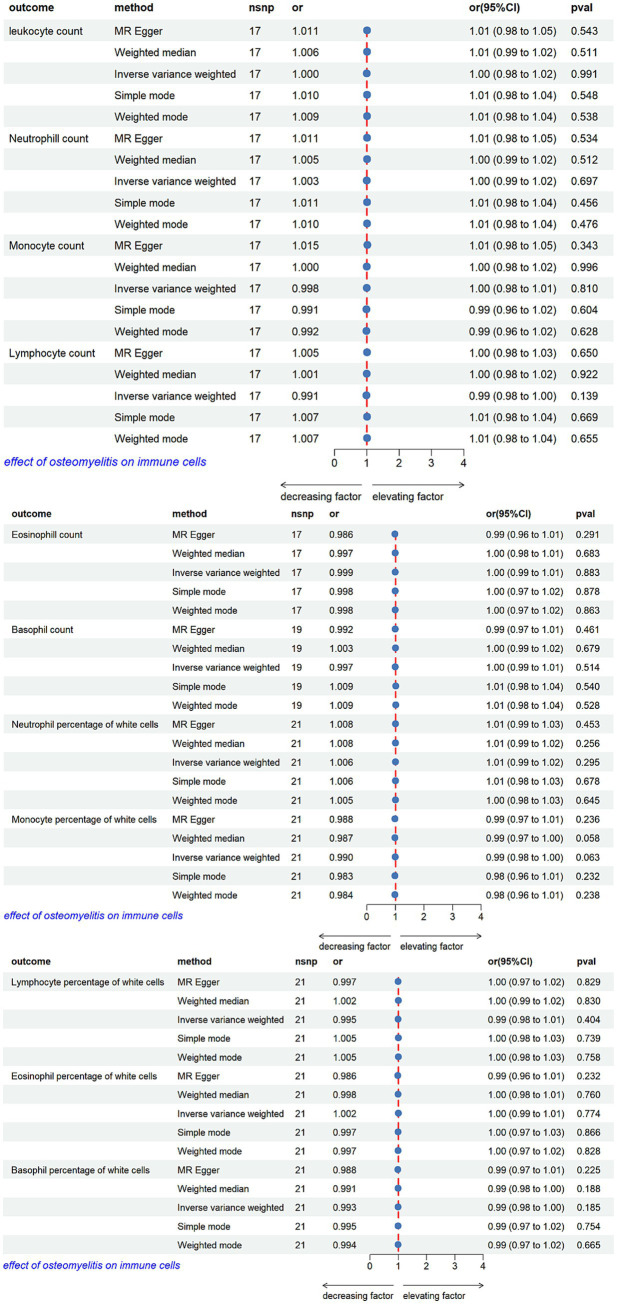
Mendelian randomization analysis of the causal effects of osteomyelitis on circulating immune cells.

We likewise performed reverse-direction MR (osteomyelitis to immune-cell traits) using IVW, WM, MR-Egger, weighted mode, and simple mode estimators. None of these approaches yielded evidence for a causal effect of osteomyelitis on any of the broad immune-cell categories examined (Figure 4). Horizontal pleiotropy was evaluated with the MR-Egger intercept, and heterogeneity was quantified by both IVW and MR-Egger Q statistics. Significant heterogeneity was detected for the absolute counts of leukocytes and neutrophils (IVW and MR-Egger *p* < 0.05) and for the relative percentage of eosinophils (MR-Egger *p* < 0.05), indicating variability in SNP-specific estimates. Random-effects IVW models were therefore used to accommodate this dispersion.

## 4 Discussion

The incidence of osteomyelitis resulting from accidental trauma has increased with advancements in transportation and industry. Epidemiological studies have indicated an increase in the incidence of osteomyelitis from 11.4 cases per 100,000 people in the 1970s to 24.4 cases per 100,000 in the 2000s (15). Osteomyelitis remains a clinically challenging condition. A critical factor contributing to the progression from the acute to chronic stages is the ability of bacteria to readily form biofilms within osteomyelitic lesions. These biofilms impair the immune cell recognition and hinder pathogen clearance (16). Immunotherapy is a promising approach to target bacteria embedded in biofilms. The activation of immune cells enhances their capacity to recognize and attack bacteria within the biofilm matrix, disrupting its structure and aiding in infection clearance (17, 18). Furthermore, the application of immunotherapy can reduce reliance on antibiotics. This mitigates the risk of antibiotic resistance development associated with prolonged or high-dose antibiotic use, helps preserve the efficacy of antibiotic treatments, and provides a more sustainable therapeutic strategy for osteomyelitis (17, 19, 20). Different types of immune cells play distinct roles in the immune responses to osteomyelitis. Identifying key immune cell populations is crucial for developing precise immunotherapeutic strategies, such as enhancing or modulating the functions of specific immune cells to achieve optimal therapeutic outcomes. Currently, there is a paucity of research on immune cell alterations in patients with osteomyelitis. To address this gap, we recruited patients who had undergone internal fixation removal surgery as the control group, representing a near-normal physiological state preoperatively. By analyzing routine clinical blood test data, we compared changes in absolute counts and relative percentages of various immune cells. This approach aimed to delineate specific alterations in different immune cell types associated with osteomyelitis.

Based on the bacterial culture results, osteomyelitis was categorized into four subgroups: G+, G−, mixed infections, and culture-negative. G− infections, often caused by *Staphylococcus aureus*, are characterized by high virulence, invasiveness, toxin and enzyme production, leading to bone destruction and inflammation (21). G− infections, typically caused by *Escherichia coli* and *Pseudomonas aeruginosa*, trigger inflammation via endotoxins in their cell walls and are prone to antibiotic resistance (22). Patients with mixed infections experience worsened tissue damage and inflammation owing to bacterial interactions, high resistance rates, and a tendency to relapse (2, 23). In culture-negative cases, pathogens may be inactive. During early infection, innate immune cells such as neutrophils and monocyte-macrophages rapidly migrate to eliminate bacteria. Subsequently, adaptive immune cells (T and B lymphocytes) are activated to suppress excessive inflammation and assist in pathogen clearance (12, 24). Bacterial biofilms are sessile multicellular aggregates embedded in a self-synthesized extracellular matrix that adheres to biotic or abiotic surfaces, shielding resident microbes from environmental stress and host defenses (16, 17). Within human tissues, these structures dramatically reduce the efficacy of both surgical debridement and antimicrobial therapy. Biofilms formed by different types of bacteria exhibit distinct characteristics and interact differently with immune cells. Gram-positive bacterial biofilms rely on protein and polysaccharide polymers and adhere to host cells or implanted material surfaces via cell wall-anchored surface proteins (16, 25). The immunogenicity of lipoteichoic acid in Gram-positive bacteria is relatively weaker compared to the lipopolysaccharide of Gram-negative bacteria. Biofilms of Gram-positive bacteria can induce neutrophil extracellular trap formation and secrete toxins such as leukocidins, which perforate and lyse neutrophil membranes, leading to cell death (26, 27). In contrast, Gram-negative bacterial biofilms are primarily composed of extracellular DNA, polysaccharides, and lipids, and initial attachment is mediated by pili and flagella. Polysaccharide components such as alginate produced by Gram-negative bacteria can mask surface lipopolysaccharide, preventing recognition by immune cells via Toll-like receptor 4 and thereby facilitating immune evasion (28). Additionally, Gram-negative bacteria can directly induce neutrophil necrosis through the secretion of rhamnolipids (29, 30). The damaging effects of both Gram-positive and Gram-negative biofilms on neutrophils provide a mechanistic explanation for the observed neutropenia in certain infections. Polymicrobial biofilms, often resulting from mixed infections, pose a significantly greater threat than single-species biofilms, as synergistic interactions between bacteria can lead to more severe infections and heightened immunosuppression (16, 31). Each osteomyelitis subgroup has distinct bacterial infection characteristics, necessitating a separate analysis of immune cell trends.

Our study revealed that patients with osteomyelitis exhibited significantly reduced absolute neutrophil counts and relative neutrophil percentages compared to patients undergoing implant removal, both in the overall cohort and across subgroups. Vancomycin, a glycopeptide routinely employed against osteomyelitis, has been implicated in neutropenia in 2–12% of treated patients (32). Independent of drug toxicity, the infection itself generates high circulating levels of TNF-*α* and interferons that inhibit hematopoietic stem-cell self-renewal and remodel the bone-marrow niche, leading to suppressed granulopoiesis and subsequent neutropenia (33–35). This suggests systemic immune activation in response to the inflammatory state of osteomyelitis, with immune cells mobilized via peripheral circulation to infiltrate the lesion site, resulting in elevated circulating levels. Lymphocytes exert stage-specific and context-dependent roles during osteomyelitis progression. Early infection is dominated by IL-17- and RANKL-producing T-helper cells that amplify osteoclastogenesis and bone resorption, whereas in later, more stabilized phases regulatory T cells and CD8+ subsets secrete osteoprotegerin and anti-inflammatory cytokines that restrain further bone loss and support reparative angiogenesis (36–38). Eosinophils have been shown to inhibit osteoclast function by releasing peroxidases, thereby reducing bone loss. Human studies have shown a negative correlation between the expression of the osteoclast-associated gene *ACP5* and eosinophil-specific gene *RNASE2* (39). Given that osteomyelitis involves concurrent bone destruction and repair, eosinophilia may help mitigate bone loss. Basophils can induce the differentiation and maturation of B lymphocytes (40), potentially aiding pathogen clearance during osteomyelitis. Additionally, basophils can promote local infiltration of eosinophils (41). Thus, the complex immune microenvironment within osteomyelitis lesions is intricately linked to the local processes of bone damage and repair.

Our clinical research revealed significant changes in different types of immune cells in patients with osteomyelitis compared to those in relatively healthy internal fixator patients. However, the causal relationship between immune cells and osteomyelitis remains unclear. MR exploits germline genetic variants as instrumental variables to probe—rather than definitively establish—causal relationships in observational data (42). Using summary-level statistics from two large GWAS, we performed a two-sample MR analysis at the resolution of broad immune-cell categories. Both the inverse-variance weighted (IVW) and weighted-median (WM) estimators suggested that genetically predicted higher circulating lymphocyte counts are associated with an increased risk of osteomyelitis. However, the available exposure GWAS do not dissect subset-specific effects (e.g., CD4+ vs. CD8+ T cells, regulatory B or T cells). Consequently, these findings should be viewed as hypothesis-generating rather than strong causal evidence, and cannot adjudicate the mechanistic roles of individual lymphocyte subpopulations. Higher-resolution immune-trait GWAS will be required to refine these causal hypotheses. Our findings align with those of Liu et al. (43), who reported a positive association between elevated CD8+ T-cell counts and susceptibility to osteomyelitis. These results are also consistent with the clinical results, as most osteomyelitis subgroups showed increased peripheral lymphocyte counts and percentages compared to internal fixator patients. However, patients with mixed-infection osteomyelitis exhibited no significant differences in lymphocyte counts and percentages. This may be because mixed infections require patients to combat multiple bacterial species, leading to a complex lymphocyte response with diverse cellular activation and suppression mechanisms. Bone homeostasis is maintained by a balance between osteoblasts and osteoclasts. Studies indicate that circulating B lymphocytes produce osteoprotegerin to inhibit bone loss, while activated T and B cells in inflammatory states secrete osteoclastogenic factors such as RANKL, IL-17A, and TNF-*α*. These factors promote osteoclast differentiation and maturation, resulting in bone loss (44). Thus, lymphocytes exert bidirectional regulatory effects on bone metabolism. Targeting lymphocytes may be a novel approach for future osteomyelitis immunotherapy.

Traditional management of osteomyelitis relies on surgery, antibiotics, and bone repair. However, growing antimicrobial resistance weakens antibiotic efficacy, leading to disease recurrence in patients with osteomyelitis (3–6). Immunotherapy as an adjunct to antibiotics can boost pathogen killing and infection resolution. Immune cells eliminate pathogens and regulate bone metabolism. Inflammatory osteoblasts and osteoclasts participate in the repair of bone defects in osteomyelitis (13). Thus, immunotherapies targeting immune cells may offer new avenues for treating osteomyelitis.

Our study had some limitations. Owing to the limited resolution of the exposure GWAS data, the current Mendelian randomization analysis is unable to resolve heterogeneity within lymphocyte subsets. Future MR investigations should leverage higher-resolution immune-trait GWAS to refine causal hypotheses at the single-cell-subset level. This MR study used GWAS databases, which may have population stratification biases. However, additional data from diverse regions and ethnic groups are required to confirm these results. Additionally, the clinical data were obtained from a single-center study. Multi-center studies are required for validation.

## 5 Conclusion

In summary, our study demonstrates that, compared to patients undergoing internal fixator removal, patients with osteomyelitis exhibit a distinct pattern of immune cell distribution in the peripheral circulation, characterized by a reduction in neutrophils and an increase in lymphocytes, eosinophils, and basophils. Moreover, our MR analysis indicated a potential positive association between lymphocyte levels and the risk of osteomyelitis, which was supported by subgroup analyses showing significantly elevated lymphocyte counts across most subgroups, except in cases of mixed infections. These findings not only provide valuable insights for clinicians in terms of diagnosis and prognosis but also highlight the potential of lymphocyte-targeted therapies for osteomyelitis. Further research is warranted to elucidate the specific mechanisms underlying the role of lymphocytes in the pathogenesis of osteomyelitis and to advance the goal of personalized treatment for osteomyelitis based on different pathogens.

## Data Availability

The original contributions presented in the study are included in the article/Supplementary material, further inquiries can be directed to the corresponding authors.
