# Supplementary material for: Trends in immune cell profiles of osteomyelitis: a clinical study supported by Mendelian randomization analysis
Source: Front Med (Lausanne). 2025 Sep 29;12:1669180. doi: 10.3389/fmed.2025.1669180 (PMC12515866; doi:10.3389/fmed.2025.1669180)
Supplement: Supplementary file 1 [file Table_1.docx]

**Supplementary Table 1: Baseline data analysis before and after PSM of patients with overall osteomyelitis and implant-removal**

| Items | Before matching | | | | | After matching | | | | |
| --- | --- | --- | --- | --- | --- | --- | --- | --- | --- | --- |
|  | IR (n = 378) | | OM (n = 235) | | p | IR (n = 235) | | OM (n = 235) | | *p* |
| Gender (n) | male | female | male | female | 0.002 | male | female | male | female | 0.754 |
|  | 277 | 101 | 197 | 38 |  | 195 | 40 | 197 | 38 |  |
| Age (years) | 48.5 [34, 58] | | 48 [36, 57] | | 0.907 | 46.43 ± 14.76 | | 46.50 ± 13.53 | | 0.958 |
| Height (cm) | 170 [164.75, 175] | | 170 [165, 174] | | 0.886 | 170 [165, 175] | | 170 [165, 174] | | 0.530 |
| Weight (kg) | 70 [60, 78] | | 71 [65, 80] | | 0.040 | 70 [63, 80] | | 71 [65, 80] | | 0.545 |
| Smoking (n) | yes | no | yes | no | 0.591 | yes | no | yes | no | 0.788 |
|  | 140 | 238 | 82 | 153 |  | 79 | 156 | 82 | 153 |  |
| Diabetes (n) | yes | no | yes | no | 0.947 | yes | no | yes | no | 0.405 |
|  | 36 | 342 | 22 | 213 |  | 16 | 219 | 22 | 213 |  |

IR: implant-removal; OM: osteomyelitis
