# Supplementary material for: Trends in immune cell profiles of osteomyelitis: a clinical study supported by Mendelian randomization analysis
Source: Front Med (Lausanne). 2025 Sep 29;12:1669180. doi: 10.3389/fmed.2025.1669180 (PMC12515866; doi:10.3389/fmed.2025.1669180)
Supplement: Supplementary file 2 [file Table_2.docx]

**Supplementary Table 2: Comparison of baseline characteristics between the Gram-positive monomicrobial osteomyelitis group and the implant-removal group**

| Items | IR (n = 378) | | G+ OM (n = 113) | | *p* |
| --- | --- | --- | --- | --- | --- |
| Gender (n) | male | female | male | female | 0.119 |
|  | 277 | 101 | 91 | 22 |  |
| Age (years) | 48.5 [34, 58] | | 49 [36, 58] | | 0.379 |
| Height (cm) | 170 [164.75, 178] | | 170 [164, 172] | | 0..412 |
| Weight (kg) | 70 [60, 78] | | 70 [61.5, 79] | | 0.511 |
| Smoking (n) | yes | no | yes | no | 0.625 |
|  | 140 | 238 | 39 | 74 |  |
| Diabetes (n) | yes | no | yes | no | 0.538 |
|  | 36 | 342 | 13 | 100 |  |

IFR: internal fixator removal; OM: osteomyelitis
