# Supplementary material for: Trends in immune cell profiles of osteomyelitis: a clinical study supported by Mendelian randomization analysis
Source: Front Med (Lausanne). 2025 Sep 29;12:1669180. doi: 10.3389/fmed.2025.1669180 (PMC12515866; doi:10.3389/fmed.2025.1669180)
Supplement: Supplementary file 3 [file Table_3.docx]

**Supplementary Table 3: Comparison of baseline characteristics before and after PSM between the Gram-negative monomicrobial osteomyelitis group and the implant-removal group**

| Items | Before matching | | | | | After matching | | | | |
| --- | --- | --- | --- | --- | --- | --- | --- | --- | --- | --- |
|  | IR (n = 378) | | G- OM (n = 29) | | *p* | IR (n = 29) | | G- OM (n = 29) | | *p* |
| Gender (n) | male | female | male | female | 0.001 | male | female | male | female | - |
|  | 277 | 101 | 29 | 0 |  | 29 | 0 | 29 | 0 |  |
| Age (years) | 48.5 [34, 58] | | 48.5 [39.5, 56] | | 0.752 | 44.72 ± 16.60 | | 47.55 ± 8.56 | | 0.309 |
| Height (cm) | 170 [164.75, 175] | | 170 [167.5, 175] | | 0.550 | 172.03 ± 5.60 | | 170.21 ± 5.62 | | 0.194 |
| Weight (kg) | 70 [60, 78] | | 75 [69, 80] | | 0.059 | 73.00 ± 13.99 | | 74.59 ± 11.97 | | 0.636 |
| Smoking (n) | yes | no | yes | no | 0.308 | yes | no | yes | no | 0.500 |
|  | 140 | 238 | 8 | 21 |  | 6 | 23 | 8 | 21 |  |
| Diabetes (n) | yes | no | yes | no | 0.885 | yes | no | yes | no | 1.000 |
|  | 36 | 342 | 3 | 26 |  | 2 | 27 | 3 | 26 |  |

IR: implant-removal; OM: osteomyelitis
