# Supplementary material for: Trends in immune cell profiles of osteomyelitis: a clinical study supported by Mendelian randomization analysis
Source: Front Med (Lausanne). 2025 Sep 29;12:1669180. doi: 10.3389/fmed.2025.1669180 (PMC12515866; doi:10.3389/fmed.2025.1669180)
Supplement: Supplementary file 4 [file Table_4.docx]

**Supplementary Table 4: Baseline characteristics of the polymicrobial (Gram-positive and Gram-negative) osteomyelitis group versus the implant-removal group before and after PSM**

| Items | Before matching | | | | | | | After matching | | | | | |
| --- | --- | --- | --- | --- | --- | --- | --- | --- | --- | --- | --- | --- | --- |
|  | IR (n = 378) | | | G+ & G- OM (n = 19) | | | *p* | IR (n = 19) | | | G+ & G- OM (n = 19) | | *p* |
| Gender (n) | male | | female | male | | female | 0.116 | male | | female | male | female | 1.000 |
|  | 277 | | 101 | 17 | | 2 |  | 18 | | 1 | 17 | 2 |  |
| Age (years) | 48.5 [34, 58] | | | 46 [32, 52] | | | 0.540 | 46.84 ± 18.85 | | | 44.63 ± 12.25 | | 0.691 |
| Height (cm) | 170 [164.75, 175] | | | 170 [165, 175] | | | 0.647 | 172 [170, 178] | | | 170 [165, 175] | | 0.367 |
| Weight (kg) | 70 [60, 78] | | | 70 [67, 75] | | | 0.683 | 69.79 ± 12.06 | | | 70.74 ± 11.59 | | 0.810 |
| Smoking (n) | yes | no | | yes | no | | 0.986 | yes | no | | yes | no | 1.000 |
|  | 140 | 238 | | 7 | 12 | |  | 6 | 13 | | 7 | 12 |  |
| Diabetes (n) | yes | no | | yes | no | | 0.371 | yes | no | | yes | no | 1.000 |
|  | 36 | 342 | | 3 | 16 | |  | 2 | 17 | | 3 | 16 |  |

IR: implant-removal; OM: osteomyelitis
