# Supplementary material for: Trends in immune cell profiles of osteomyelitis: a clinical study supported by Mendelian randomization analysis
Source: Front Med (Lausanne). 2025 Sep 29;12:1669180. doi: 10.3389/fmed.2025.1669180 (PMC12515866; doi:10.3389/fmed.2025.1669180)
Supplement: Supplementary file 5 [file Table_5.docx]

**Supplementary Table 5: Comparison of baseline characteristics before and after PSM between the culture-negative osteomyelitis group and the implant-removal group**

| Items | Before matching | | | | | | | After matching | | | | | |
| --- | --- | --- | --- | --- | --- | --- | --- | --- | --- | --- | --- | --- | --- |
|  | IR (n = 378) | | | OM (n = 69) | | | *p* | IR (n = 69) | | | OM (n = 69) | | *p* |
| Gender (n) | male | | female | male | | female | 0.167 | male | | female | male | female | 1.000 |
|  | 277 | | 101 | 56 | | 13 |  | 55 | | 14 | 56 | 13 |  |
| Age (years) | 48.5 [34, 58] | | | 45 [32.5, 56] | | | 0.371 | 48.39 ± 15.89 | | | 44.64 ± 14.53 | | 0.155 |
| Height (cm) | 170 [164.75, 175] | | | 170 [166, 174] | | | 0.963 | 169.12 ± 7.49 | | | 169.25 ± 7.16 | | 0.908 |
| Weight (kg) | 70 [60, 78] | | | 75 [65, 80] | | | 0.019 | 70 [62.5, 80] | | | 75 [65, 80] | | 0.560 |
| Smoking (n) | yes | no | | yes | no | | 0.899 | yes | no | | yes | no | 0.839 |
|  | 140 | 238 | | 25 | 44 | |  | 23 | 46 | | 25 | 44 |  |
| Diabetes (n) | yes | no | | yes | no | | 0.161 | yes | no | | yes | no | 1.000 |
|  | 36 | 342 | | 3 | 66 | |  | 3 | 66 | | 3 | 66 |  |

IR: implant-removal; OM: osteomyelitis
