# Supplementary material for: Trends in immune cell profiles of osteomyelitis: a clinical study supported by Mendelian randomization analysis
Source: Front Med (Lausanne). 2025 Sep 29;12:1669180. doi: 10.3389/fmed.2025.1669180 (PMC12515866; doi:10.3389/fmed.2025.1669180)
Supplement: Supplementary file 6 [file Table_6.docx]

**Supplementary Table 6: Characteristics of studies included in the MR analysis of the causal effects of immune cells on osteomyelitis**

| Trait | GwasID | Sample size | NO. of SNPs | Population | Author | NO. Of IVs | F |
| --- | --- | --- | --- | --- | --- | --- | --- |
| Osteomyelitis | ieu-b-4975 | 486484 | 12243512 | European | Hamilton F | - | - |
| leukocyte count | ukb-d-30000_irnt | 350470 | 13586282 | European | Neale lab | 264 | 29.61~960.11 |
| neutrophil cell count | ieu-b-34 | 563946 | - | European | Vuckovic, D | 396 | 12.15~1619.84 |
| Monocyte count | ebi-a-GCST90018967 | 349856 | 19052644 | European | Sakaue S | 272 | 21.86~2156.35 |
| Lymphocyte count | ebi-a-GCST90018962 | 349861 | 13586283 | European | Sakaue S | 279 | 23.34~850.09 |
| Eosinophil counts | ebi-a-GCST90018953 | 349856 | 19022494 | European | Sakaue S | 262 | 14.05~729.35 |
| Basophil count | ebi-a-GCST90025997 | 438607 | 4232896 | European | Barton AR | 30 | 26.94~193.98 |
| Neutrophil percentage of white cells | ebi-a-GCST90002399 | 408112 | 40312502 | European | Vuckovic D | 293 | 28.25~550.46 |
| Monocyte percentage of white cells | ebi-a-GCST90002394 | 408112 | 40309733 | European | Vuckovic D | 339 | 27.26~3428.77 |
| Lymphocyte percentage of white cells | ebi-a-GCST90002389 | 408112 | 40312257 | European | Vuckovic D | 293 | 28.31~633.71 |
| Eosinophil percentage of white cells | ebi-a-GCST90002382 | 408112 | 40312065 | European | Vuckovic D | 333 | 27.49~1795.98 |
| Basophil percentage of white cells | ebi-a-GCST90002380 | 408112 | 40311711 | European | Vuckovic D | 117 | 30.04~1030.18 |
