# Supplementary material for: Trends in immune cell profiles of osteomyelitis: a clinical study supported by Mendelian randomization analysis
Source: Front Med (Lausanne). 2025 Sep 29;12:1669180. doi: 10.3389/fmed.2025.1669180 (PMC12515866; doi:10.3389/fmed.2025.1669180)
Supplement: Supplementary file 7 [file Table_7.docx]

**Supplementary Table 7: Characteristics of studies included in the MR analysis of the causal effects of osteomyelitis on immune cells**

| Trait | GwasID | Sample size | NO. of SNPs | Population | Author | NO. Of IVs | F |
| --- | --- | --- | --- | --- | --- | --- | --- |
| Osteomyelitis | ieu-b-4975 | 486484 | 12243512 | European | Hamilton F | - | - |
| leukocyte count | ebi-a-GCST90013976 | 396624 | 10783695 | European | Mbatchou J | 17 | 2223.54~2656.85 |
| neutrophil cell count | ebi-a-GCST90013984 | 395949 | 10783686 | European | Mbatchou J | 17 | 2223.54~2656.85 |
| Monocyte count | ebi-a-GCST90013983 | 395949 | 10783686 | European | Mbatchou J | 17 | 2223.54~2656.85 |
| Lymphocyte count | ebi-a-GCST90013982 | 395949 | 10783686 | European | Mbatchou J | 17 | 2223.54~2656.85 |
| Eosinophil counts | ebi-a-GCST90013985 | 395949 | 10783686 | European | Mbatchou J | 17 | 2223.54~2656.85 |
| Basophil count | ebi-a-GCST90018946 | 349856 | 19026124 | European | Sakaue S | 19 | 2223.54~2656.85 |
| Neutrophil percentage of white cells | ebi-a-GCST90002399 | 408112 | 40312502 | European | Vuckovic D | 21 | 2223.54~3762.81 |
| Monocyte percentage of white cells | ebi-a-GCST90002394 | 408112 | 40309733 | European | Vuckovic D | 21 | 2223.54~3762.81 |
| Lymphocyte percentage of white cells | ebi-a-GCST90002389 | 408112 | 40312257 | European | Vuckovic D | 21 | 2223.54~3762.81 |
| Eosinophil percentage of white cells | ebi-a-GCST90002382 | 408112 | 40312065 | European | Vuckovic D | 21 | 2223.54~3762.81 |
| Basophil percentage of white cells | ebi-a-GCST90002380 | 408112 | 40311711 | European | Vuckovic D | 21 | 2223.54~3762.81 |
