# Supplementary material for: Trends in immune cell profiles of osteomyelitis: a clinical study supported by Mendelian randomization analysis
Source: Front Med (Lausanne). 2025 Sep 29;12:1669180. doi: 10.3389/fmed.2025.1669180 (PMC12515866; doi:10.3389/fmed.2025.1669180)
Supplement: Supplementary file 8 [file Data_Sheet_1.PDF]

G*Power 3.1.9.2

File Edit View Tests Calculator Help

Central and noncentral distributions | Protocol of power analyses

[6] -- *Monday, July 07, 2025 -- 03:11:55*

t tests – Means: Difference between two dependent means (matched pairs)

**Analysis**:	Post hoc: Compute achieved power		
**Input**:	Tail(s)	=	Two
	Effect size dz	=	0.5
	α err prob	=	0.05
	Total sample size	=	235
**Output**:	Noncentrality parameter δ	=	7.6648549
	Critical t	=	1.9701536
	Df	=	234
	Power (1–β err prob)	=	1.0000000

Test family: t tests

Statistical test: Means: Difference between two dependent means (matched pairs)

Type of power analysis: Post hoc: Compute achieved power – given α, sample size, and effect size

Input Parameters

Tail(s)	Two
Effect size dz	0.5
α err prob	0.05
Total sample size	235

Determine =>

Output Parameters

Noncentrality parameter δ	7.6648549
Critical t	1.9701536
Df	234
Power (1–β err prob)	1.0000000

X–Y plot for a range of values

Calculate

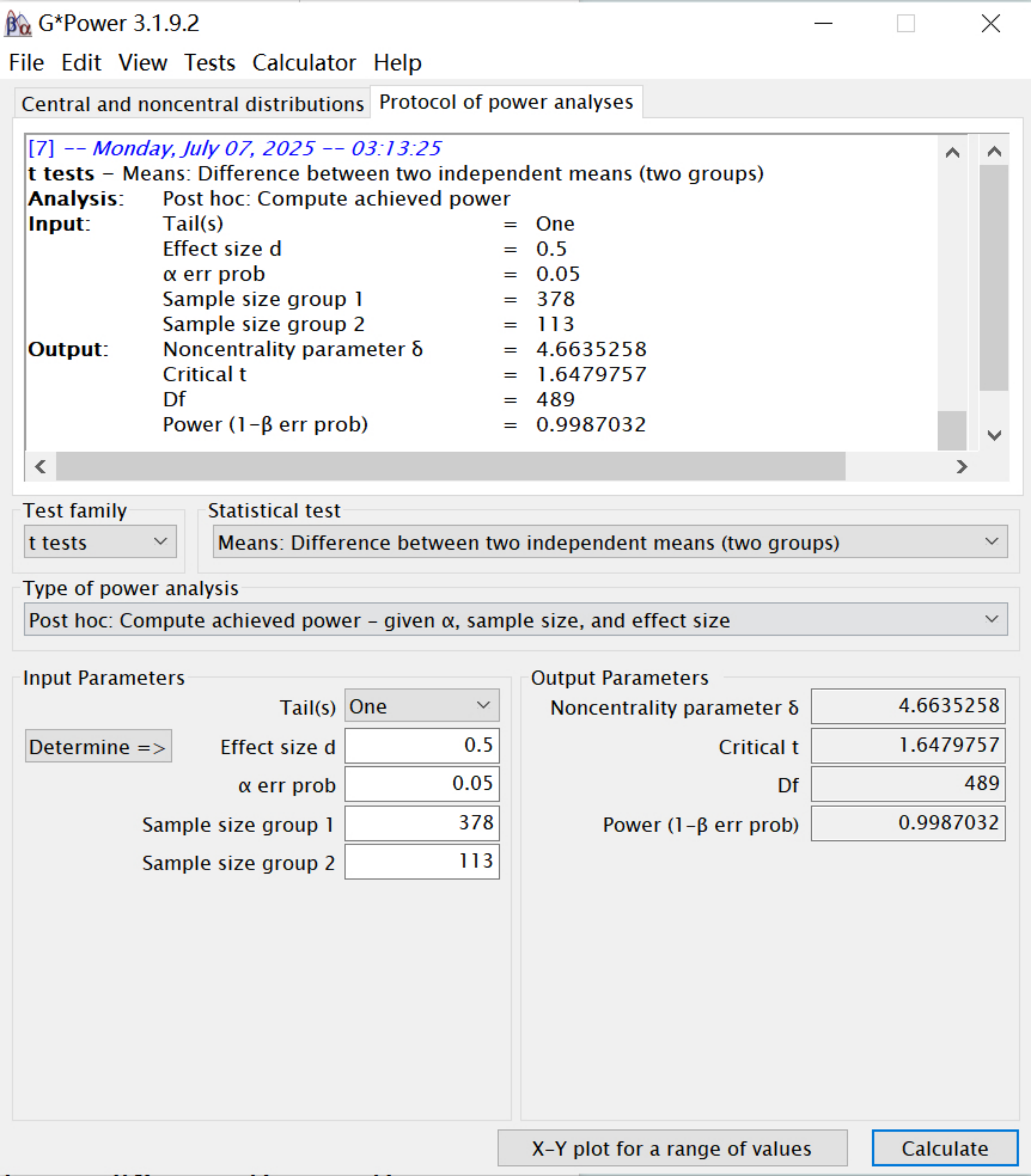
G*Power 3.1.9.2
File Edit View Tests Calculator Help
Central and noncentral distributions
Protocol of power analyses
[7] -- Monday, July 07, 2025 -- 03:13:25
t tests – Means: Difference between two independent means (two groups)
Analysis: Post hoc: Compute achieved power
Input: Tail(s) = One
Effect size d = 0.5
α err prob = 0.05
Sample size group 1 = 378
Sample size group 2 = 113
Output: Noncentrality parameter δ = 4.6635258
Critical t = 1.6479757
Df = 489
Power (1–β err prob) = 0.9987032
Test family
t tests
Statistical test
Means: Difference between two independent means (two groups)
Type of power analysis
Post hoc: Compute achieved power – given α, sample size, and effect size
Input Parameters
Tail(s) One
Determine =>
Effect size d 0.5
α err prob 0.05
Sample size group 1 378
Sample size group 2 113
Output Parameters
Noncentrality parameter δ 4.6635258
Critical t 1.6479757
Df 489
Power (1–β err prob) 0.9987032
X–Y plot for a range of values
Calculate

G*Power 3.1.9.2

File Edit View Tests Calculator Help

Central and noncentral distributions | Protocol of power analyses

[8] -- *Monday, July 07, 2025 -- 03:14:06*

t tests – Means: Difference between two dependent means (matched pairs)

**Analysis:**	Post hoc: Compute achieved power		
**Input:**	Tail(s)	=	Two
	Effect size dz	=	0.5
	α err prob	=	0.05
	Total sample size	=	29
**Output:**	Noncentrality parameter δ	=	2.6925824
	Critical t	=	2.0484071
	Df	=	28
	Power (1–β err prob)	=	0.7386963

Test family: t tests

Statistical test: Means: Difference between two dependent means (matched pairs)

Type of power analysis: Post hoc: Compute achieved power – given α, sample size, and effect size

Input Parameters

Tail(s)	Two
Effect size dz	0.5
α err prob	0.05
Total sample size	29

Determine =>

Output Parameters

Noncentrality parameter δ	2.6925824
Critical t	2.0484071
Df	28
Power (1–β err prob)	0.7386963

X–Y plot for a range of values

Calculate

G*Power 3.1.9.2

File Edit View Tests Calculator Help

Central and noncentral distributions | Protocol of power analyses

[9] -- Monday, July 07, 2025 -- 03:14:41

t tests – Means: Difference between two dependent means (matched pairs)

**Analysis**:	Post hoc: Compute achieved power		
**Input**:	Tail(s)	=	Two
	Effect size dz	=	0.5
	α err prob	=	0.05
	Total sample size	=	19
**Output**:	Noncentrality parameter δ	=	2.1794495
	Critical t	=	2.1009220
	Df	=	18
	Power (1−β err prob)	=	0.5408630

Test family: t tests

Statistical test: Means: Difference between two dependent means (matched pairs)

Type of power analysis: Post hoc: Compute achieved power – given α, sample size, and effect size

Input Parameters

Tail(s)	Two
Effect size dz	0.5
α err prob	0.05
Total sample size	19

Determine =>

Output Parameters

Noncentrality parameter δ	2.1794495
Critical t	2.1009220
Df	18
Power (1−β err prob)	0.5408630

X-Y plot for a range of values

Calculate

G*Power 3.1.9.2

File Edit View Tests Calculator Help

Central and noncentral distributions | Protocol of power analyses

[10] -- *Monday, July 07, 2025 -- 03:15:17*

t tests – Means: Difference between two dependent means (matched pairs)

**Analysis**:	Post hoc: Compute achieved power		
**Input**:	Tail(s)	=	Two
	Effect size dz	=	0.5
	α err prob	=	0.05
	Total sample size	=	69
**Output**:	Noncentrality parameter δ	=	4.1533119
	Critical t	=	1.9954689
	Df	=	68
	Power (1–β err prob)	=	0.9835793

Test family: t tests

Statistical test: Means: Difference between two dependent means (matched pairs)

Type of power analysis: Post hoc: Compute achieved power – given α, sample size, and effect size

Input Parameters

Tail(s)	Two
Effect size dz	0.5
α err prob	0.05
Total sample size	69

Determine =>

Output Parameters

Noncentrality parameter δ	4.1533119
Critical t	1.9954689
Df	68
Power (1–β err prob)	0.9835793

X-Y plot for a range of values | Calculate
